# Drying Characteristics and Microbiological Quality Assessment of Solar-Dried Tomato

**DOI:** 10.1155/2022/2352327

**Published:** 2022-10-28

**Authors:** Mavis Owureku-Asare, Ibok Oduro, Firibu K. Saalia, Charles Tortoe, Jonathan Ampah, Kingsly Ambrose

**Affiliations:** ^1^Department of Agricultural and Biological Engineering, Purdue University, West Lafayette, Indiana, USA; ^2^Department of Food Science and Technology, Kwame Nkrumah University of Science and Technology, Kumasi, Ghana; ^3^School of Engineering, University of Ghana, Accra, Ghana; ^4^Food Research Institute, Council for Scientific and Industrial Research, Accra, Ghana

## Abstract

Tomato (*Lycopersicon esculentum*) is an important vegetable used in cooking most local foods in Ghana. At the peak season of harvesting, high loses are incurred because of the absence of tomato processing facilities to store, process, and extend the shelf life of fresh tomatoes. Solar drying has been proven to be a more efficient and low-cost method of enhancing quality and adding value to tomato and other vegetables. However, there are concerns about the functionality and quality of the dried products by consumers due to the methods of drying used. In this study, a passive mixed-mode solar dryer suitable for drying tomato was adapted and used to investigate the dehydration characteristics and microbiological quality of the dried tomato. The efficiency of a passive solar dryer was evaluated and used in the processing of fresh tomato to powder. The processing involved the pretreatment of 6 mm slices of fresh Roma variety of tomato by dipping in potassium metabisulfite solution and ascorbic acid solution. The moisture content, moisture ratio, and dehydration rate of solar-dried tomato were assessed. The 24 h dryer efficiency of 24.2% facilitated the drying process of tomato (final moisture content of 12-14%). Aerobic mesophile counts were lower in solar-dried tomato pretreated with potassium metabisulfite (3.90 CFU/g) compared with sun-dried samples (4.85 CFU/g). Solar-dried tomato powder is safer for consumption compared with open sun-dried tomato samples.

## 1. Introduction

Tomato (*Lycopersicon esculentum*) is an important vegetable used in cooking most local foods in Ghana [1]. Tomato production is a significant economic activity with annual production of 366,772,000 tonnes per annum [2] at the height of the harvest season in Ghana. In the coastal areas of West Africa, tomato production is seasonal, since most of the production is rainfed, and there is limited availability of heat-tolerant varieties for off-season production [3, 4]. This seasonality leads to a severe shortage of the produce during the dry season and prices skyrocket, compromising tomato stability and accessibility for consumers. On the contrary, there is glut during the rainy season, and in most cases, farmers abandon their fields when prices are so low [5].

Attempts to solve the problem of seasonal glut and scarcity through commercial processing have not been successful [6]. At the peak season of harvesting tomato, losses of between 20 and 50% are incurred due to the absence of tomato processing setups or facilities, which results in fluctuations in tomato prices [7].

Open sun drying is a widely practiced method of processing agricultural products. However, it is a slow process and may cause color degradation, poor rehydration, high microbial growth, and loss of certain nutrients [8]. Different solar dryer designs have been proposed to overcome the shortcomings of open sun drying. However, two designs, viz., cabinet and tunnel type dryers, have been proposed for domestic applications, as they require no electric power and can be used in rural areas [9–11]. Comparative studies by Forson [12] and Mohamad [13] on natural convection solar crop dryer designs revealed that the mixed-mode natural convection solar crop dryer (MNCSCD) is the most promising and effective dryer suited for tropical humid areas. The MNCSCD is a cabinet type of solar dryer which consists of a transparent cover and a solar air heater. It allows for natural airflow and utilizes direct solar energy and the convective energy of heated air to dry food in a drying area or chamber.

The efficiency and performance of dryers are influenced by environmental variables such as the solar radiation, ambient temperature, airflow, and the ambient relative humidity [14, 15]. These factors must be considered in solar dryer design for tomato as they influence the quality (sensory and nutritional parameters and rehydration capacity) of dried tomato products [16]. The lack of appropriate evaluation procedures for solar dryers has sometimes resulted in selecting the wrong dryer type and operating conditions for certain food products [17]. Various studies have been conducted on the thermal efficiency of solar dryers [16, 18–20]. However, there is no general agreement on methodology to compare their performance [21]. This is partly because food products have different drying rates, which are dependent on prevailing environmental conditions. Solar energy varies with time and geographic location, which makes it difficult to compare results obtained from solar dryers even if they are of the same type. In rural Ghana and in many tropical rural communities around the world where there is poor access to the national electric grid, MNCSCD is ideal for processing dried tomato and saving considerably on electric energy [14, 22, 23]. Hence, adapting and enhancing dryer design to improve airflow by natural convection will enhance in the drying of tomato.

The overall drying efficiencies of solar dryers have been shown to vary widely depending on the loading densities and weather conditions [24]. The time required to decrease a product moisture content to 15% is an indicator of the dryer efficiency [16]. Also, in the design of passive solar dryers for vegetables, an efficiency of 12.5% was reported by Leon et al. [16] while natural convection solar crop dryer's efficiency ranging from 10% to 15% was reported by Forson et al. [25].

Inasmuch as drying under direct sunlight has the advantages of low to zero energy cost and nonreliance on grid powered electricity, there are attributable disadvantages. The process is slow and labour intensive, and products may easily be contaminated by dust, insects, and other pests [26]. In a bid to address these challenges, the current solar dryer was constructed using local materials. The confined nature of food samples within the cabinet served as a mechanism that prevented food loss as compared to drying under open sun. Also, pebbles were introduced into the collector compartment with intent to improve heat absorption and retention. Assessing the efficiency of the natural convection dryer for drying tomato, for conditions in Ghana, will be useful for farmers and small-scale industries since this will form the basis for selection of an appropriate dryer and suitable drying conditions for processing tomato. The objectives of this study are to evaluate the efficiency of a mixed-mode natural convection solar dryer, to assess the drying characteristics, and to evaluate the microbial quality of pretreated, solar-dried tomato.

## 2. Materials and Methods

### 2.1. Sample Preparation

Fresh tomato (Roma variety) was purchased from a farmer in Bolgatanga and transported in wooden crates at ambient temperature to the laboratory in Accra. Tomato was stored in an air-conditioned room at 16°C. Ripe but firm tomato was selected and washed under running tap water and with 1% sodium metabisulfite solution. Tomato was cut into slices of 5 mm, using a tomato slicer (Jaccard stainless steel mandolin, USA). This size was selected based on the results from the preliminary studies. The initial moisture content of tomato was measured using the air-oven method [27].

### 2.2. Pretreatments prior to Dehydration Process

Sliced tomato was divided into three parts and assigned to three treatments as follows: dipping in (a) 1% potassium metabisulfite (KMS) solution for 10 minutes and (b) 1% ascorbic acid (1 : 1) and (c) the untreated be kept as control.

### 2.3. Drying

**Figure 1:**
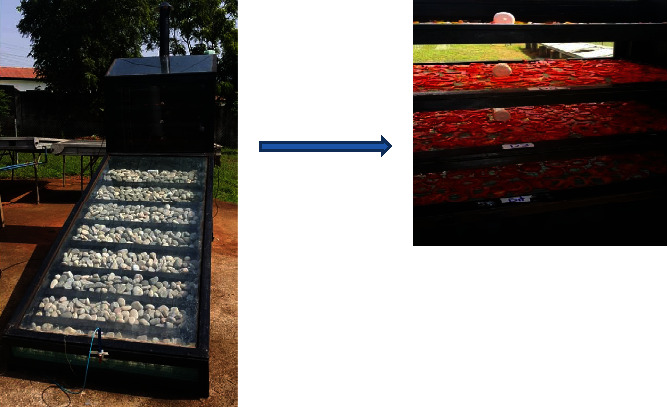
Solar cabinet dryer.

**Figure 2:**
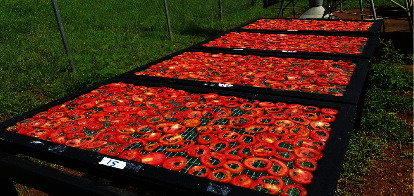
Cut tomato on drying racks in open sun drying.

The study evaluated the performance of a mixed-mode natural convection solar dryer (MNCSD) (Figure 1) designed for drying tomatoes. Open sun drying method (Figure 2) was used as a control for comparison purposes.

#### 2.3.1. Drying Equipment

Drying experiments were performed in a prototype solar cabinet dryer. The dimensions of the dryer chamber were length: 1 m, width: 0.6 m, and height: 1 m. A solar dryer with a collector tilt angle of 15.6° facing south north position for optimum solar radiation in Accra, Ghana, is located at 49 m above sea level at 5.6301 N 0.1801 W (accuracy: 3 m radius, device info: Garmin eTrex 30).

### 2.4. Description of Dryer

**Figure 3:**
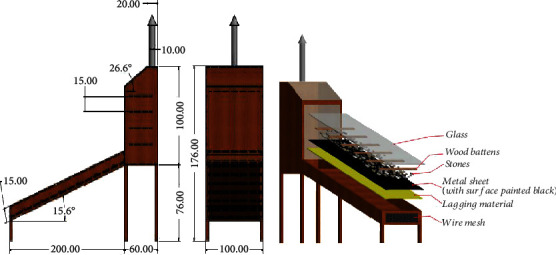
Schematic representation of mixed-mode solar cabinet dryer for tomato.

A passive mixed-mode solar dryer (PMSD) was designed at the CSIR-Food Research Institute, Accra, based on the known construction of solar cabinet dryers [28, 29]. The dryer framework including the collector was made of treated redwood. The physical properties of the dryer are captured in Table 1. The drying chamber housed 8 racks, each of dimensions 870 mm × 530 mm made of plastic mesh fastened on wooden frames and related to a drying capacity of 5 kg/tray. The dryer comprised of three main components, namely, the primary and secondary collector and the drying chamber, in which the crop to be dried is placed.  Figure 3 represents a schematic of the solar dryer designed using Autodesk® Inventor® 2016 (Build 200138000, 138). A dryer was constructed from 25.4 mm thick plywood, 1.5 mm thick metal sheet, 25.4 mm spaced wire mesh, sieving material, and 5 mm thick glass sheet. A collector was constructed using local materials of wood, aluminum sheets, metals, fiber glass, stones, and glass sheet. The collector was insulated by placing 10 mm thick glass wool insulation between the wooden base and metal sheet. 25.4 mm × 50.8 mm wooden battens were then positioned at intervals of 250 mm from the opening of collector. These battings served as restrictions for stones which were placed in the primary collector to facilitate heat absorption and release into the drying chamber. To enable flow of convective heat and prevent insects going into the drying chamber, pieces of wire mesh and sieving material were used to close up the opening of the primary collector. The drying chamber has a loading capacity of 4 kg for thin layer drying of tomato slices. An access door to the drying chamber is located at the rear. An exit air vent located on top of the secondary collector facilitates removal of moist air from the drying chamber. The interior of the dryer was painted with food grade black paint for maximum absorption of solar radiation. Pebbles weighing roughly 96 kg were distributed uniformly throughout the collector area.

### 2.5. Dehydration Processes

Pretreated tomato slices (4 kg) were uniformly spread on rectangular mesh trays (87 cm × 53 cm) and placed in the drying chamber of the solar dryer. The weight of samples for moisture content analysis was recorded every two hours by a digital balance of 0.001 g accuracy (Scaltec Instruments, Gottingen, Germany). Samples were taken from the solar dryer, weighed, and placed in a hot air oven set at 105°C for 10 hr. Drying progressed till moisture content of samples reached 13-15% moisture content for solar-dried samples. Experiments were replicated three times.

### 2.6. Monitoring of Process Variables: Humidity, Wind Speed, and Temperature

#### 2.6.1. Solar Dryer

Probes connected to a data logger (Hobo U23 Pro V2, USA) were placed at five different locations in the solar dryer chamber (4 placed on the drying racks and 1 in the solar collector). Data for temperature and relative humidity were recorded at a one-minute interval using a Lab VIEW signal express program and exported to Microsoft Office Excel for further analysis. Thermocouple (ALMEMO 2890-9, Germany) measuring software WinControl and stored data on SD card was used to measure the temperature of the outlet and inlet air of the dryer.

Inlet wind speed was recorded by ALMEMO digital vane anemometer FVAD 15S220 (Germany). Airflow outlet was measured by thermos-anemometer probe FVAD 35 TH5K2 (Germany). Solar radiation was measured at a 10 min interval using a solar radiation sensor (silicon pyranometer sensor S-LIB-M003). Three replicated experiments were carried out between 9:30 and 16:30 h on sunny days (using British Broadcasting Corporation (BBC) weather forecast). At the end of each day of drying, the tomato samples were packed into impermeable polythene bags wrapped with aluminum foil and stored in a refrigerator at 10°C.

#### 2.6.2. Sun Drying

In order to compare the performance of the cabinet dryer with that of open sun drying, 4 kg of sliced tomato was placed on drying trays (similar to that used in the solar dryer) as seen in plate 2. Ambient air temperature was measured using thermocouple sensor NiCR-Ni (Germany) and wind velocity using ALMEMO 2890-9 (Germany). Solar insolation was measured at 10 min interval using a solar radiation sensor (silicon pyranometer sensor S-LIB-M003). Triplicate sun drying experiments were carried out simultaneously with a cabinet solar dryer. Sun drying progressed till moisture content of samples reached 19-23% moisture.

#### 2.6.3. Moisture Content

Moisture content of tomato samples was determined by using the standard methodology [27]. 3 g of tomato samples was placed in a metal dish (preweighed) and placed in the air-oven (Gallenkamp, United Kingdom) for 8 h at 105°C. The dish with dried sample was cooled in a desiccator, and the average moisture content from triplicate samples was determined.

#### 2.6.4. Moisture Ratio (MR)

In thin layer drying, the moisture ratio during drying was calculated as follows:

MR was calculated using the following equation:

(1) $$\begin{array}{c} \text{MR}=\frac{M-M_{\text{e}}}{M_{0}-M_{\text{e}}}, \end{array}$$

where MR is the dimensionless moisture ratio, *M* is the moisture content at time *t*, and *M*_o_ and *M*_e_ are the initial and equilibrium moisture contents, respectively, on dry basis. During thin layer drying of tomato slices in the cabinet dryer, the samples were not exposed to uniform relative humidity and temperature continuously. So, the moisture ratio was simplified according to Schirra et al. [30] and Doymaz [31] to

(2) $$\begin{array}{c} \text{MR}=\frac{M}{M_{0}}. \end{array}$$

#### 2.6.5. Solar Dryer Efficiency (SDE)

The efficiency of a solar drying system can be defined as the ratio of heat energy utilized in the vaporization of the moisture from food samples to that of solar radiation incident on the collector and the crop surface [32]. The efficiency of a solar dryer is a measure of how effectively the input energy to the drying system is used in drying food samples. For natural convection solar dryers, the overall dryer efficiency (SDE) can be calculated using [33]

(3) $$\begin{array}{c} \text{SDE}=\frac{M\times L}{I\times A\times t}, \end{array}$$

where *M* is the mass of moisture removed (kg), *L* is the latent heat of vaporization of water, *I* is the average solar radiation over the drying period (W/m^2^), *A* is the area of collector (m^2^), and *t* is the drying time (s).

The average initial and final moisture contents of food samples in the present study were considered in calculating mass of moisture removed (*M*). The latent heat of vaporization (*L*) of water was assumed to be 2.26 MJ/kg. The average daily solar radiation (*I*) recorded during the drying period ranged between 100 and 1000 W/m^2^.

The type of dryer, product dried, weather, and final moisture level are only a few of the variables that affect drying efficiency overall. With longer drying times comes a significant reduction in drying efficiency. Solar drying efficiency was found to typically range between 10-15% for natural convection dryers and 20-30% for forced convection dryers [34].

#### 2.6.6. Drying Rate (DR)

The weight of samples in the dehydrator was recorded every two hours and drying rate calculated using equation (4) and curves plotted with the values obtained.

(4) $$\begin{array}{c} \text{DR}=\left(\frac{\text{Difference in weight for time between reading }\left(\text{g}\right)}{\text{Time interval }\left(\min\right)}\right). \end{array}$$

#### 2.6.7. Microbiology Analyses of Tomato Powder

*(1) Homogenization and Serial Dilution*. For all solid samples, ten (10) grams was added to 90.0 ml sterile salt peptone solution (SPS) containing 0.1% peptone and 0.8% NaCl, with pH adjusted to 7.2 and homogenized in a stomacher (Lad Blender, Model 4001, Seward Medical), for 30 s at normal speed. From appropriate tenfold dilutions, 1 ml aliquot of each dilution was directly inoculated into sterile Petri dish plates and the appropriate media added for enumeration and isolation. All analyses were done in duplicate.

*(2) Enumeration of Aerobic Mesophiles*. Aerobic mesophiles were enumerated by the pour plate method using plate count agar medium (Oxoid CM 325; Oxoid Ltd., Basingstoke, Hampshire, UK). Plates were incubated at 30°C for 72 hr in accordance with the Nordic Committee on Foods Analysis Method (NMKL. No. 86, 2006).

*(3) Enumeration of Yeasts and Molds*. Yeasts and molds were enumerated by the pour plate method using oxytetracycline-glucose yeast extract agar (OGYEA) (Oxoid CM 545; Oxoid Ltd., Basingstoke, Hampshire, UK) to which OGYEA supplement was added to suppress bacteria growth. The pH was adjusted to 7.0 and incubated at 25°C for 3-5 d in accordance with ISO 7954 (1987).

*(4) Enumeration and Isolation of Total Coliform*. Coliform bacteria were counted by the pour plate method using tryptone soya agar medium (Oxoid CM 131) and adjusted to pH 7.3 and overlaid with violet red bile agar (Oxoid CM 107) with pH adjusted to 7.4 and incubated at 37°C for 24 hours. Colonies were confirmed using brilliant green bile broth (Oxoid CM 31) at pH of 7.4 and incubated at 37°C for 24 hours (NMKL no. 44, 2004). Positive reaction was indicated by the production of gas at the entire bent portion of the Durham tube.

*(5) Enumeration of Escherichia coli*. E. coli bacteria were enumerated by the pour plate method using tryptone soya agar medium (Oxoid CM 131) adjusted to the pH 7.3 and overlaid with violet red bile agar (Oxoid CM 107) with pH adjusted to 7.4 and incubated at 44°C for 24 hours. Suspected colonies were confirmed using E.C. broth (Oxoid CM 853) with pH adjusted to 6.9. Colonies that produced gas that has filled the entire concave part of the Durham tube were taken as thermos-tolerant coliform bacteria. To determine E. coli, thermo-tolerant bacteria were confirmed for indole production. This was done by subculturing into positive tubes into tryptone broth and incubated at 44°C for 24 hours. An indole test was done by adding 0.3-0.5 ml of Kovac's reagent into the culture. Red ring coloration at the surface of tryptone broth indicated indole positive (NMLK no. 125, 2005).

### 2.7. Data Analysis

Data were analysed using Microsoft Office, Excel 2017 and Minitab version 7. Means and standard deviations of the data were presented. Graphs were generated using Microsoft Office, Excel 2017.

## 3. Results and Discussion

### 3.1. Drying Performance of Solar Dryer

**Figure 4:**
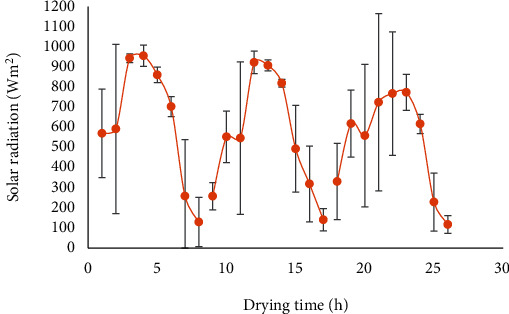
Variation in solar irradiation during the drying of tomato slices.

The contribution of environmental variables such as the solar radiation, ambient temperature, and the ambient relative humidity is considered important in solar dryer design. The average daily variation of solar radiation ranged from 116.85 to 955.6 W/m^2^ over the drying hours for sun drying and solar drying of tomato (Figure 4). Both sun and solar drying utilized 26 h drying period. Also, the drying time for all the treatments was 8-9 per day.

**Figure 5:**
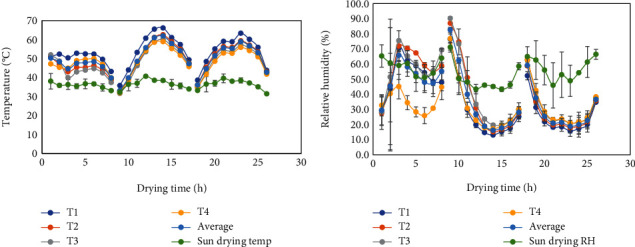
Variation in temperature (a) and relative humidity (b) of sun and solar during the drying of tomato slices. T1: top tray; T2: second tray; T3: third tray; T4: bottom tray in the drying chamber.

The drying temperature reached a maximum average of 62°C for a solar dryer while the maximum ambient temperature was 41°C for sun drying, with the top tray recording a maximum temperature of 66°C (Figure 5(a)). The mean temperature ranged recorded was 30-41°C for sun drying and 36-66°C for solar drying. It is ideal to dry tomato between 55 and 60°C to reduce case hardening [35]. Tomato slices with initial moisture content of 95-96% were reduced to 14-15% final moisture content for solar-dried tomato and 19-22% for sun-dried tomato over 23-25 h. Lower relative humidity recorded in the solar dryer compared to that of the ambient air may have facilitated the faster drying rate of tomato slices in the solar dryer (Figure 5(b)).

During experimentation, solar radiation may be measured as instantaneous or a daily average. In the course of data collection, conditions of cloud cover during drying may have contributed to variations in solar radiation. Studies conducted by Chemkhi et al. [36] provide evidence of fluctuations during solar drying of agricultural crops. They recorded high solar radiation above 600 W/m^2^ from the onset (10 am to 2 pm) of drying, and it gradually declined below 600 W/m^2^ after 3 pm. Similar data was recorded while observing samples under open sun and greenhouse drying [37]. Hourly fluctuations were observed right from onset till completion of the drying process within a 10-hour period. Higher relative humidity values recorded under open sun compared to solar drying may have also contributed to variations in solar radiation because increase in air humidity reduces its capacity to absorb moisture from the drying fruit [38] and displaces the quality of incident solar rays.

**Figure 6:**
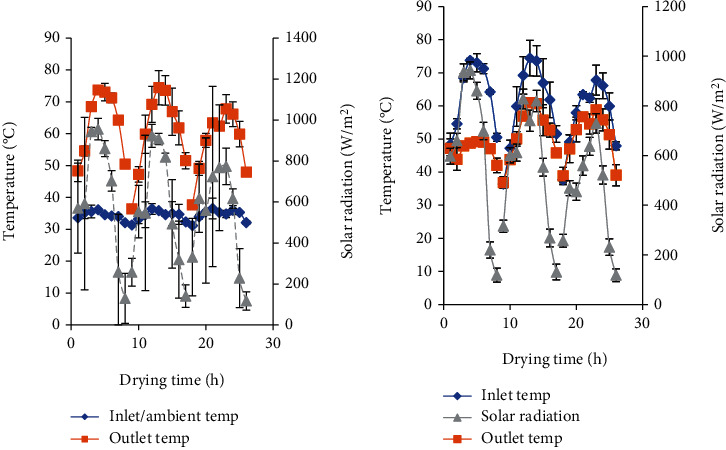
Variation in temperature of (a) solar dryer collector and (b) solar drying chamber.

The area of the collector receiving solar radiation is an important consideration in the estimation of drying efficiency for the mixed-mode dryer as tomato slices received solar radiation indirectly from the primary collector and directly from the secondary collector. The primary and secondary collectors indicate the total solar radiation collection area. The average inlet (ambient) temperature of air entering the collector was 31-37°C, and outlet temperature leaving the collector and entering the drying chamber of the solar dryer ranged from 47 to 74°C during the drying period (Figure 6).

An elevation in temperature of at least 10–15°C from the ambient is required for effective drying to take place, and this is a useful indication of the collector/dryer performance [16, 39]. The drying temperature (outlet air from collector and inlet air into the drying chamber) reached a maximum of 74°C while the maximum ambient temperature was 36°C, a difference of 38°C which is significantly high and gives an indication of the relatively high efficiency of the dryer. Temperature of outlet air leaving the drying chamber ranged between 37 and 61°C, which is significantly high and could be channeled and recycled as an alternative heat source.

Temperatures in the range of 50-60°C are recommended for drying temperature-sensitive products like fruits and vegetables [16]. However, temperatures up to 65°C may be used at the beginning but should be lowered as food begins to dry and should not exceed 55°C in the last hour of drying as this may affect the quality of tomato and cause case hardening or browning of tomato [40]. The maximum temperature entering the drying chamber was 74°C; however, the maximum temperature recorded in the drying chamber of the dryer was 66°C at 1 pm in the afternoon on the top drying rack (directly under the secondary collector). The temperature of air entering the chamber varied with the intensity of sun radiation, and the time of the day as such the high temperature of 66°C recorded was for a short period.

### 3.2. Solar Dryer Efficiency (SDE)

Efficiency of the solar cabinet dryer markedly varied with the moisture content of the product and the incident solar irradiation over the drying period. For a passive solar dryer, the temperature generated was not constant and varied during the drying period. Due to this variation in temperature, the overall efficiency is lower in passive dryers than active solar dryers where the temperature of the dryer can be regulated. The first-day efficiency is important for the drying process since the moisture content of tomato is the highest on the first day and an inefficient drying system during this period compromises the quality of dried tomato [20]. The high moisture of the tomato at this phase promotes the growth of microorganism which makes tomato susceptible to spoilage.

Global radiation is composed of direct and diffuse radiation. The direct solar radiation is the component which comes directly from the sun. The diffuse radiation component is produced when the direct sun rays are dispersed into all directions by the various molecules and particles in the atmosphere. The amount of diffuse radiation is influenced by the climatic and geographic conditions. Clouds and atmospheric conditions (such as haze and dust layers over big cities) all have a significant impact on the proportion of diffuse radiation and ultimately on global radiation [28]. Lower radiation values resulted in poor absorption and subsequently lower drying rates. For drying to occur within a specified period, a regular amount of heat is required from direct radiation. From the drying efficiency formula, time plays a critical role in calculating efficiencies of drying systems. Intermittent and inconsistent solar rays contribute to two of the downsides of solar drying: longer drying times and poor drying rates. Hence, lower solar radiation may be the result of diffuse radiation, cloud cover, and dust layers, reducing drying rates and culminating into lower drying efficiencies.

Solar radiation from the onset of drying (in the early morning) is usually low. These are the first incident rays hitting the food samples and therefore require some time to heat up moisture in the samples. Relative humidity in the mornings is also higher compared to that in the afternoons. This is from the effect of cool temperatures during the night. Hence, for drying to take effect, incident solar heat must be able to overcome the low morning temperature (high RH) in order to build up heat within the food samples. The gradual heat build-up causes the RH in the solar dryer to decrease faster compared to that within the environment. Drop in RH results in commencement of the drying process.

From the mathematical formula applied in determining drying efficiency, it can be concluded that efficiency is directly proportional to mass of moisture removed (kg), latent heat of vaporization of water, the average solar radiation over the drying period (W/m^2^), and drying time (s). The higher efficiency for day 1 compared to that for day 2 is due to the removal of larger quantities of moisture from tomatoes on day 1 compared to day 2. Assuming all other parameters such as latent heat and time remained constant for both days, mass of moisture removed and solar radiation would serve as the major influencers on efficiency. Solar radiation recorded on day 1 begun from around 580 W/m^2^ and increased to 950 W/m^2^ within 4 h, after which it gradually decreased to 250 W/m^2^ at 7 h. Radiation on day 2 however begun at 250 W/m^2^, increased to 900 W/m^2^ at 4 h and gradually decreased to 300 W/m^2^. Day 1 received slightly higher solar radiation and also removed much larger moisture from tomato compared to day 2, consequently influencing a higher efficiency for day 1.

**Figure 7:**
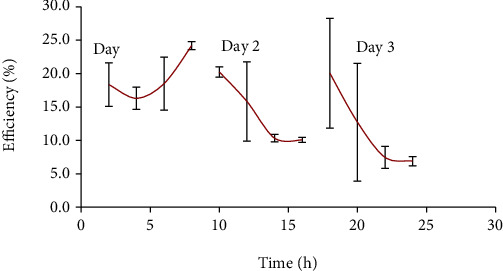
Changes in solar dryer efficiency during drying period.

The onset of drying recorded 18% efficiency, and this increased to 24% at the end of the drying period on day 1 (Figure 7). Due to the large quantity of moisture removed from tomatoes on day 1, day 2 recorded little moisture loss culminating into lower efficiency at the end of drying. Day 3 recorded an even much lower efficiency compared to day 2 because all of the free moisture was removed. What then remained was the internal bound moisture which is attached to the biological matter. Research by Prasad et al. [41] using a solar-biomass dryer operating for 1.5 days recorded 28.57% efficiency using 8 mm turmeric rhizome slices as test samples. Again, for 8 mm ginger slices dried in a hybrid solar dryer, efficiency ranging from 13 to 18% was recorded. The indirect-type natural convection solar dryer with integrated solar collector-storage and biomass-backup heaters designed by Madhlopa and Ngwalo [42] recorded efficiencies between 11 and 13%. Studies on a drying test rig documented maximum overall efficiency of the drying system as 21.24% [43]. The efficiency values documented in previous studies were found to corroborate that recorded in the current research.

### 3.3. Drying Characteristics of Tomato Slices

**Figure 8:**
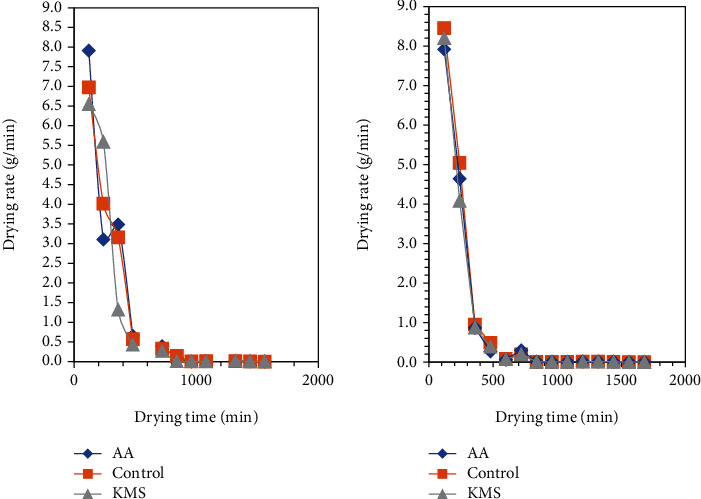
Drying rate of (a) solar- and (b) sun-dried tomato slices during drying. AA: pretreated tomato with 1% ascorbic acid solution; KMS: pretreated tomato slices with 1% potassium metabisulfite; control: no pretreatment.

Figure 8 shows the drying rate of solar- and sun-dried tomato over drying period. The constant phase was not observed in both solar and sun drying of tomato, while three falling rate periods were observed for solar-dried tomato. The falling rate period is usually the longest part of a drying operation, and in some foods, the falling rate period is the only part of the drying curve to be observed [44].

Three falling phases were observed in all pretreated solar-dried samples. The overall drying rate on the first day of drying was faster and steeper (depicted in the drying curve in Figure 8(b)) for sun drying samples. However, a higher moisture content (19-20%) was achieved for sun-dried tomato over the drying period. The drying rate of fruits can also be improved by pretreatments such as blanching and chemical treatment before drying [31, 45]. Drying rate at the falling phase was facilitated by the removal of unbound water from the surface of tomato slices on the first day of drying. The drying rate curves indicate that both sun and solar drying mainly occurred during the falling phase, similar to drying of pretreated and fresh, preosmosed, blanched, and sulfited food samples in studies by other researchers for pumpkin slices [46], red chili [47], carrots [31], and okra [48] where no constant rate was observed during drying. This indicated that diffusion is the main mechanism for moisture movement in dried tomato [49].

The moisture content of tomato expresses the total amount of water (free water, adsorbed water, and water of hydration) present [50]. It is difficult to monitor drying operation of solar dryers closely till the product final moisture content reaches the same value in solar dryers because the rate of removal of water in the drying process is highly dependent on ambient conditions such as temperature and relative humidity [20]. However, for comparative evaluation of dryers, the final moisture content of dried tomato at a specific given time can be used to evaluate and compare the performance of dryers under evaluation [16]. The moisture content of solar-dried tomato decreased from 95% to 14-15% for pretreated samples, which is optimal to preserve the products. Samples pretreated with KMS lost water faster than the control and samples pretreated with ascorbic acid. The moisture content of sun-dried tomato at 26 hours ranged between 19 and 20%. It was difficult to attain lower final moisture content for sun-dried tomato because of erratic changes in relative humidity of ambient air it was exposed to which directly affected the moisture content. Since dried tomato is hygroscopic in nature, it tends to absorb water from the ambient air when humidity increases as temperature of the drying medium fall. In a similar experiment by Rajkumar et al. [51], the drying methods and the time taken to dry tomato slices were lower in a vacuum solar dryer than in open sun drying. The decrease in drying time was mainly due to the higher vapor pressure gradient created in the vacuum, which facilitated the removal of moisture from the sample.

**Figure 9:**
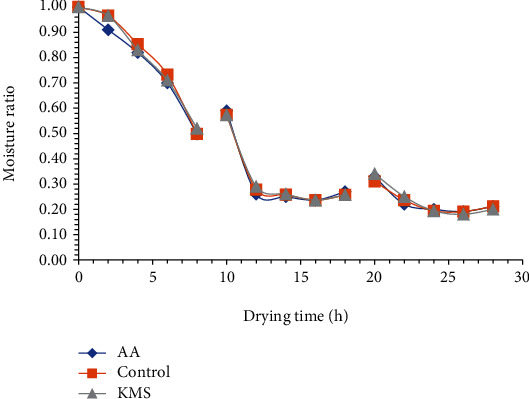
Variations in moisture ratio of solar-dried tomato slices during drying.

**Figure 10:**
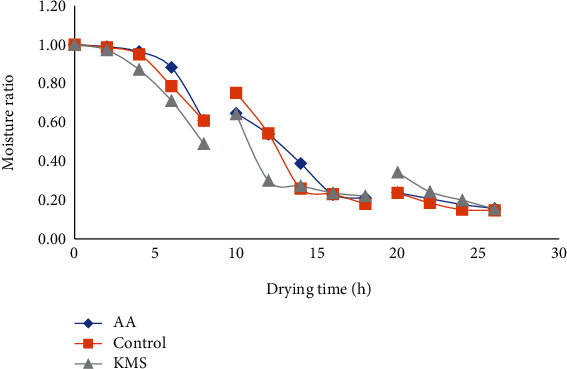
Variations in moisture ratio of sun-dried tomato slices over drying period. AA: pretreated tomato with 1% ascorbic acid solution; KMS: pretreated tomato slices with 1% potassium metabisulfite; Control: no pretreatment.

The moisture ratio versus drying time for solar and sun tomato slices is shown in Figures 9 and 10, respectively. The moisture ratio decreased continuously with drying time for solar- and sun-dried tomato. The moisture ratio also decreased with increasing drying air temperature and time in the fresh and pretreated pumpkin [46]. The drying of tomato slices occurs in the falling phase, and no constant rate was observed similar to drying behavior reported for tomato [51, 52], red chillies [53], and onion slices [54]. The continuous decrease in moisture ratio during the falling phase period is an indication of the internal mass transfer which occurred by diffusion of moisture from the internal tissues of tomato tissues.

### 3.4. Microbiological Quality of Tomato Powder

Table 2 shows the microbiological quality of tomato powder. The yeast (2.48 log CFU/g) and mold (2.30 log CFU/g) counts were below the allowable limit of 3.0 log CFU/g for yeast and 4.0 log CFU/g for molds, set by the International Commission for microbiological specifications for foods (ICMS). Pretreatment aids in the inhibition of enzymatic browning and reduces water activity and microbial growth [16, 55, 56] which results in minimal quality degradation. Yeast and mold counts were significantly (*p* ≤ 0.05) lower for solar-dried tomato pretreated with KMS than for samples pretreated with ascorbic acid and the control. Osmophilic yeasts are of no public health significance, but they are responsible for spoilage and development of off or fermented odors, which limit shelf life [57].

Significant differences in yeast count were observed between the control and pretreated tomatoes (*p* ≤ 0.05). In a study by Latapi and Barrett [58], sun-dried tomatoes pretreated with sodium metabisulfite did not show signs of spoilage or off-odors and had lower yeast counts than those not treated with sodium metabisulfite which had reduced yeast growth. Latapi and Barrett [58] recommended pretreatment of 6% or 8% sodium metabisulfite concentrations for 5 minutes to control yeast growth for tomato. With untreated sun-dried tomatoes, yeast counts were 4.9 log CFU/g exceeding allowable limits (10^3^/g). This resulted in fermented odors with physical signs of spoilage, and yeast growth was reduced significantly (3.5 log CFU/g) when tomatoes were dipped in a 10% salt solution for 5 min before sun drying [58]. *E. coli* was not detected in any of the pretreated dried samples. Aerobic mesophile counts were also lower in solar-dried tomato pretreated with KMS compared to sun-dried samples. Yeast (4.20 log CFU/g) counts recorded for sun-dried tomato pretreated with KMS was higher than the set limits. Sun-dried samples pretreated with ascorbic acid also recorded yeast and molds within the acceptable allowable ICMS set limits.

Microbial counts were expressed as base-10 logarithms of colony forming units per gram (log CFU/g) for solar- and sun-dried tomato. Counts were the same for both control and treated samples (10 CFU/g).

### 3.5. Effect of Sulfur Dioxide on Microbial Load of Tomato

The residual sulfur dioxide content of both solar- and sun-dried tomato samples with potassium metabisulfite was within safe limit of <2000 ppm (Table 2). Pretreating tomato with 1% KMS implies an initial concentration of 3400 ppm of sulfur dioxide before drying, but this was significantly reduced to 740.8 ppm for solar-dried tomato and 480.55 ppm for sun-dried tomato [59]. The heating process removes sulfites by decomposing the sulfites and subsequent removal of the resulting free sulfur dioxide gas [60]. A positively strong correlation between sulfur dioxide 740.9 ppm concentration and microbial load was observed for solar-dried tomato.

Davis et al. [61] recommended an initial sulfur dioxide content of 3000 mg/kg for dried fruits to enhance the microbial safety. The safety of sulfites in foods and its alleged roles in causing certain allergic reactions and asthma have been questioned. This led to the revocation of the generally recognized as safe (GRAS) levels of sulfites for use in fresh fruits and vegetables by the FDA in 1986. Thus, most countries accept a maximum legal limit of 2000 ppm of sulfur dioxide in fruits [62]. During three months of storage of sun-dried tomato, significant losses of about 70% sulfur dioxide content were observed in sun-dried tomato, lowering the levels of sun-dried tomato. Similar results were also observed whereby higher losses of sulfur dioxide occurred in for sun-dried tomatoes with initial high concentration of 4000 ppm [63].

## 4. Conclusion

The mixed-mode solar dryer adapted for drying tomato in this study recorded a first-day efficiency of 24.2% which was highly significant in enhancing its performance in reducing the final moisture content of 14-15% for solar-dried tomato compared to 19-20% for sun-dried tomato. Data from this research supports the use of mixed-mode solar dryers compared to sun drying due to the higher drying efficiencies recorded. Most of the drying process of tomato occurred in the falling phase which enhanced drying of tomato slices. Pretreatment with potassium metabisulfite influenced the drying rate of solar-dried tomato by speeding up the drying rate in the first falling phase of dehydration. Aerobic mesophile counts were also lower (within internationally acceptable range of 3.0 log CFU/g of aerobic mesophiles count) in solar-dried tomato pretreated with potassium metabisulfite compared to sun-dried samples. E. coli was not detected in both solar- and sun-dried tomatoes. The microbiological quality for solar-dried pretreated with potassium was of good quality with low level residue of sulfur dioxide which is desirable for consumption.

## Figures and Tables

**Table 1:** Physical properties of materials for construction of solar dryer.

No.	Item	Dimension	Thermal diffusivity (m^2^/s)	Thermal conductivity (W/(m K))
1	Chimney	0.1 m diameter 0.4 m height	8.418 × 10^−5^	205
2	Plywood	1 m × 1 m × 0.6 m	8.2 × 10^−8^	0.12
3	Glass wool	0.01 m	—	0.04
4	Metal sheet	2 m × 1 m	8.418 × 10^−5^	205
5	Glass sheet	2.02 m × 1.02 m × 1 m	3.4 × 10^−7^	0.96

**Table 2:** Microbial log CFU/g counts for pretreated solar- and sun-dried tomatoes.

Drying method	Pretreatment	Aerobic Mesophiles	Molds	Yeasts	Coliform	E. coli	Sulfur dioxide (ppm dwt)
Solar	Control	5.73	3.60	3.60	3.2	0	533.32
	KMS	3.90	2.30	2.48	0	0	740.99
	AA	5.15	4.78	5.11	0	0	538.54

Sun	Control	5.00	3.48	5.02	0	0	474.47
	KMS	4.85	3.60	4.20	2.95	0	480.55
	AA	4.08	2.90	2.85	0	0	567.47

## Data Availability

The data used to support the findings of this study are available from the corresponding author upon request.
